# CTSL loss leads to anti-PD-1 immunotherapy resistance in lung cancer by suppressing the anti-tumor function of peripheral CD8^+^ T cells

**DOI:** 10.3389/fimmu.2026.1863563

**Published:** 2026-06-10

**Authors:** Ying Wang, Jing Huang, Xueyang Zou, Jiancheng Xu

**Affiliations:** Department of Laboratory Medicine, Infectious Diseases and Pathogen Biology Center, The First Hospital of Jilin University, Changchun, China

**Keywords:** CTSL, immunotherapy, NLRP3, non-small cell lung cancer, peripheral CD8^+^ T cells, resistance

## Abstract

**Background:**

Non-small cell lung cancer (NSCLC) has a high incidence rate, and most patients develop resistance to anti-PD-1 immunotherapy, resulting in shortened survival. Current evidence suggests that peripheral T cells, particularly CD8^+^ T cells, play a key role in the response to anti-PD-1 immunotherapy. However, the key molecules that impair peripheral CD8^+^ T cell function and thereby drive resistance to anti-PD-1 immunotherapy remain unclear. This study aims to demonstrate that loss of cathepsin L (CTSL) expression in peripheral CD8^+^ T cells is a critical factor driving resistance to anti-PD-1 therapy in NSCLC.

**Methods:**

Using flow cytometry, we tracked the dynamic expression patterns of CTSL in peripheral CD8^+^ T cells from NSCLC patients receiving anti-PD-1 therapy, as well as its longitudinal distribution across different T cell subsets. We then investigated the association between CTSL expression levels in peripheral CD8^+^ T cells and the expression of anti-tumor effector molecules. Finally, through bioinformatics analysis, flow cytometry, ELISA, and pharmacological interventions, we explored the functional relationship between CTSL and NLRP3 inflammasome activation in mediating the anti-tumor function of CD8^+^ T cells.

**Results:**

CTSL expression in peripheral CD8^+^ T cells was demonstrated as a predictor of improved clinical response to anti-PD-1 immunotherapy in NSCLC (*p < 0.01*). Subset analysis revealed that in anti-PD-1-resistant patients, CTSL expression was significantly reduced in effector memory CD8^+^ T cells and terminally differentiated effector memory CD8^+^ T cells. Mechanistically, CTSL upregulation enhanced the expression of functional molecules in CD8^+^ T cells, including perforin, granzyme, IFN-γ, and Ki67. Functional exploration experiments further showed not only a positive correlation between CTSL and NLRP3 expression in peripheral CD8^+^ T cells, but also that activation of NLRP3 reversed the anti-tumor dysfunction of CD8^+^ T cells induced by CTSL inhibition.

**Conclusion:**

Loss of CTSL expression in peripheral CD8^+^ T cells is a key factor driving resistance to anti-PD-1 immunotherapy in NSCLC. CTSL loss may impair the anti-tumor function of CD8^+^ T cells by inhibiting NLRP3 inflammasome activation. This study not only provides a potential circulating biomarker for predicting response to anti-PD-1 therapy, but also offers new perspectives for understanding the mechanisms of resistance.

## Introduction

Non-small cell lung cancer (NSCLC) is one of the most common malignant tumors with high incidence (1). Since most patients with advanced NSCLC are no longer eligible for surgical resection, anti-tumor immunotherapy, such as anti-programmed cell death-1 (PD-1) immunotherapy, is often used in clinical practice (2). However, some patients show no response to anti-PD-1 immunotherapy, while others who initially respond experience disease relapse over time, indicating the development of therapeutic resistance (3). Therefore, elucidating the underlying mechanisms is crucial for achieving precise patient stratification, developing resistance-related biomarkers, and designing rational therapeutic strategies to improve the efficacy of anti-PD-1 immunotherapy.

It should be noted that the core mechanism of anti-PD-1 immunotherapy lies in reactivating the anti-tumor immune response, particularly by restoring the cytotoxic function of CD8^+^ T cells to effectively eliminate malignant tumors (4). Previously, it was widely believed that anti-PD-1 immunotherapy restores T cell function within the tumor microenvironment (TME) (5). However, recent studies of T cell dynamics suggest that the T cell response required for anti-PD-1 immunotherapy may originate outside the tumor and depend more on the recruitment of peripheral T cells (6). Therefore, increasing attention has been paid to the relationship between the functional status of peripheral blood T cells, especially CD8^+^ T cells, and the outcomes of anti-PD-1 immunotherapy (7, 8). Studies have shown that integrating the phenotypic and molecular features of CD8^+^ T cells can help distinguish responders from non-responders to anti-PD-1 immunotherapy (9). For example, an increase in the proportion of peripheral Ki-67^+^CD8^+^ T cells after anti-PD-1 antibody administration can be used to identify patients who respond to treatment (10, 11). In patients with durable clinical benefit, the proportion of effector memory (CD27^+^CD28^+^CD45RA^−^CCR7^−^) CD8^+^ T cells at baseline is higher than that in patients resistant to anti-PD-1 immunotherapy (12). Moreover, upregulation of genes related to T cell receptor signaling and differentiation (such as TCF7, FOS, JUN, NFKBIZ, CD74, TNF, and CD83) in CD8^+^ T cells at baseline is positively correlated with favorable treatment response following PD-1 blockade (13). Collectively, these studies reflect the potential of molecular features of peripheral blood CD8^+^ T cells as non-invasive biomarkers for predicting response and resistance to anti-PD-1 immunotherapy.

Cathepsin L (CTSL) was initially defined as a lysosomal proteolytic enzyme and a core member of the cysteine protease family, primarily involved in protein degradation (14). Numerous studies have confirmed that CTSL is significantly overexpressed in various solid tumors, including ovarian, breast, prostate, lung, gastric, and pancreatic cancers, and that its high expression is closely associated with tumor cell metastasis (15–18). Interestingly, CTSL is also widely involved in various physiological processes, such as immune cell development, immune responses, and apoptosis (19–21). For example, impaired CTSL activity hinders the degradation of the invariant chain in thymic epithelial cells, thereby affecting MHC class II antigen presentation and leading to impaired thymic generation of CD4^+^ T cells (22, 23). In addition to its role in thymic selection, CTSL also appears to be involved in the regulation of peripheral immune homeostasis, as evidenced by the increased number of regulatory T cells in CTSL mutant mice (24). Notably, this enzyme promotes the differentiation of human CD4^+^ T cells toward the Th1 phenotype via the complement C3-interferon-gamma (IFN-γ) axis (25). In CD8^+^ T cells, lysosomal CTSL specifically cleaves and activates the perforin precursor, serving as a key molecular weapon for target cell clearance (26). These studies indicate that CTSL plays important roles in both T cell immunity and tumor development. However, how CTSL participates in the response to anti-PD-1 immunotherapy in NSCLC through peripheral blood CD8^+^ T cells has not yet been reported. Elucidating this association and exploring its mechanisms would facilitate precise patient stratification, the development of resistance-related biomarkers, and the design of rational therapeutic strategies to improve the efficacy of anti-PD-1 immunotherapy.

Our study found that loss of CTSL expression in peripheral CD8^+^ T cells is a key factor driving therapeutic resistance to anti-PD-1 therapy in NSCLC, and that measuring CTSL expression levels in peripheral CD8^+^ T cells can predict patient responses to anti-PD-1 immunotherapy. We observed that patients resistant to anti-PD-1 immunotherapy exhibited marked CTSL loss in both effector memory and terminally differentiated effector memory T cell subsets. CTSL enhances CD8^+^ T cell activity by promoting the expression of key effector molecules (perforin, granzyme B, IFN-γ, and Ki67), but does not significantly regulate interleukin-1β (IL-1β) or CD69. Through a multi-platform approach combining bioinformatics and experimental strategies, we preliminarily identified NLRP3 inflammasome activation as an important mechanism by which CTSL promotes T cell activation. These results support a model in which CTSL loss in specific CD8^+^ T cell subsets blocks NLRP3 signaling, impairs T cell function, and ultimately leads to anti-PD-1 resistance. These findings provide a potential circulating biomarker for predicting response to anti-PD-1 therapy and offer new insights into the mechanisms of immunotherapy resistance.

## Materials and methods

### Patient enrollment

To investigate the biological mechanisms underlying differential responses to anti-PD-1 therapy, a defined patient cohort was established. Eligible patients with stage III/IV NSCLC (including recurrent disease) were enrolled from the First Hospital of Jilin University between November 2024 and November 2025. Patients presenting with other malignancies or significant comorbid conditions that could confound immunological analysis were systematically excluded. All patients received conventional treatments, with individualized regimens determined by their clinicians. The anti-PD-1 antibody was given intravenously every three weeks, continuing until either disease progression or unacceptable toxicity occurred. Prior to any study procedure, written informed consent was obtained from all participants, and the study protocol received approval from the Institutional Human Ethics Committee (24k285-001).

### Response evaluation

Based on the Response Evaluation Criteria in Solid Tumors (RECIST) version 1.1, patients were stratified into responder (R) and non-responder (NR) groups. Responders was defined by a sustained clinical benefit, evidenced by a complete response (CR), partial response (PR), or stable disease (SD) persisting for over 24 weeks following anti-PD-1 immunotherapy. Conversely, non-responders were those who experienced progressive disease (PD) within the first 24 weeks of therapy.

### Clinical sample collection

We prospectively collected peripheral blood from NSCLC patients immediately before initiating anti-PD-1 therapy and after one course (week 3). Peripheral blood mononuclear cells (PBMCs) were isolated from patient blood samples by density gradient centrifugation using Ficoll-Paque (Sigma-Aldrich, Cat#10771). The isolated PBMCs were subsequently analyzed by flow cytometry.

### Cell culture

We focused on how CTSL influences peripheral CD8^+^ T cells after anti-PD-1 therapy. Specifically, we selected PBMCs from patients with NSCLC for *in vitro* culture. PBMCs were resuspended with RPMI-1640 medium (Hyclone, USA) containing 10% heat-inactivated fetal bovine serum (Thermo Fisher Scientific, USA). PBMCs from the same patient were divided into four equal portions and cultured under different conditions: 1) Control (DMSO), 2) the CTSL inhibitor Z-FY-CHO (40uM, MedChemExpress, USA), 3) the NLRP3 agonist BMS-986299 (20uM, MedChemExpress, USA), 4) Z-FY-CHO (40uM) + BMS-986299 (20uM). After 24 h of cultivation, the cells were harvested for subsequent flow cytometry analysis. Following centrifugation at 3000 rpm for 10 minutes, cell culture supernatants were collected and stored at −80 °C for subsequent IL-1β quantification by ELISA.

### Flow cytometry analysis

Flow cytometric analysis was performed on PBMCs and cell cultures using a 10-color, three-laser FACSCanto™ system (BD Bioscience, USA). Data analysis was conducted with BD FACSDiva™ software (BD Bioscience, USA) to evaluate the expression of cell surface markers (CD3, CD8, CD45RA, CCR7, and CD69), intracellular proteins (CTSL, Perforin, Granzyme B, NLRP3, IFN-γ and IL-1β), and nuclear proteins (Ki67). All antibodies used in this study were commercially sourced from R&D Systems, BD Biosciences, or Santa Cruz Biotechnology, and a detailed listing is provided in the **Supplementary Table 1**. To detect intracellular cytokines, PBMCs or cultured cells were stimulated for 4 hours at 37 °C with BD Leukocyte Activation Cocktail in the presence of BD GolgiPlug (BD Biosciences, Cat# 550583). Cells were subsequently fixed and permeabilized using the BD Transcription Factor Buffer Set (BD Biosciences, Cat# 562574) prior to intracellular staining. The gating strategies for identifying specific cell subsets are illustrated in the corresponding figures.

### ELISA for IL-1β

The level of IL-1β in the supernatant were measured with human IL-1β enzyme-linked immunoassay kit (Huabio, China) according to the manufacturer’s protocol. The optical density (450 nm) was read on a Multiskan FC microplate reader (Thermo Scientific, USA), and IL-1β concentrations were calculated based on the provided standard curve (0–1000 pg/ml).

### Analysis of immunotherapy response differential genes

To investigate the relationship between CTSL expression levels in peripheral CTLs and patient response to anti-PD-1 therapy, we retrieved the GSE111414 dataset from the GEO database (https://www.ncbi.nlm.nih.gov/geo/). This study utilized a dataset of PBMCs obtained from NSCLC patients at baseline and 4 weeks after initiating anti-PD-1 immunotherapy (27). CD8^+^ T cells were isolated from these PBMCs, and their transcriptome sequencing was performed using the Illumina HiSeq 3000 high-throughput sequencing platform. The cohort included 5 responders and 5 non-responders, as classified by RECIST v1.1. Additionally, by merging multiple non-small cell NSCLC biopsy sequencing datasets (GSE126044, GSE135222, GSE166449), we further evaluated the association between CTSL and NLRP3 expression levels in the TME and prognosis following anti-PD-1 therapy.

Based on the RNA-seq data from the above NSCLC patients, differential gene expression analysis between groups was performed using the DESeq2 package (R v4.5.0). The thresholds for identifying DEGs in RNA gene expression data should be *p <0.05* and |log2 fold change|>1. The results were visualized using the ggplot2 package. Subsequently, functional annotation and pathway enrichment analysis of the obtained differentially expressed genes were conducted using the Metascape platform (28). Finally, target genes were determined based on an area under the receiver operating characteristic curve value ≥ 0.7.

### Statistical analysis

The normality of continuous variables was assessed using the Shapiro-Wilk test. Parametrically distributed data were analyzed by the Student’s t-test, while non-parametric data were analyzed using the Mann-Whitney U test (two groups) or the Kruskal-Wallis test (multiple groups). We used Fisher’s exact test to assess associations between categorical variables, given our small sample size. In the figures, significance is denoted by asterisks: *p < *0.05*, **p < *0.01*, and ***p < *0.001*. We further evaluated whether the proportion of CD8^+^CTSL^+^ T cells in peripheral blood could predict treatment resistance by constructing receiver operating characteristic (ROC) curves. The optimal cutoff value derived from the ROC analysis was then used to stratify patients into high-risk and low-risk groups of resistance to anti-PD-1 immunotherapy. Progression-free survival (PFS) between these two groups was visualized and compared using Kaplan-Meier curves. All statistical analyses were carried out with GraphPad Prism 10.0 (RRID: SCR_002798) and SPSS 27.0 (RRID: SCR_002865).

## Results

### CTSL loss in peripheral CD8^+^ T cells after anti-PD-1 immunotherapy was a poor prognostic signature

**Figure 1:**
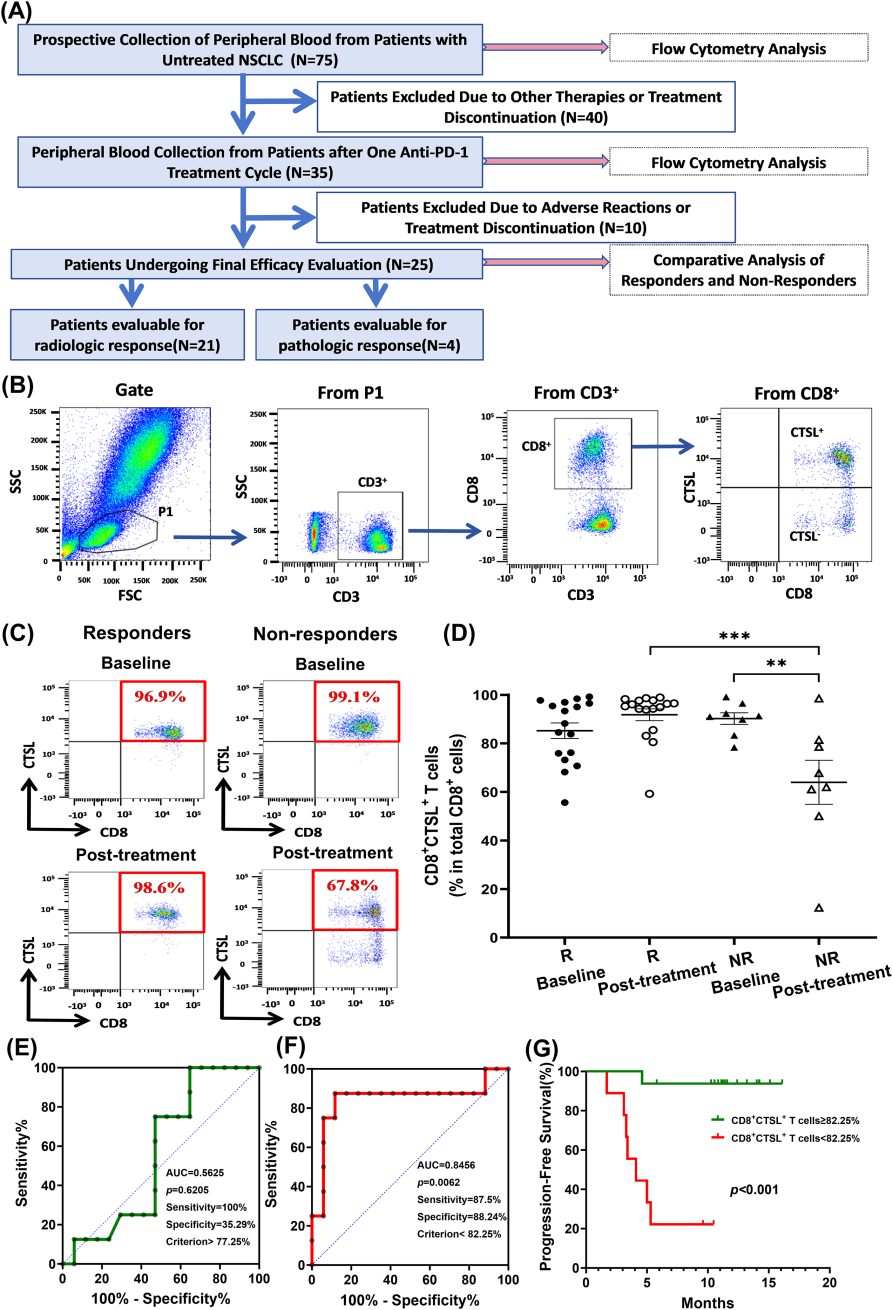
CTSL loss in peripheral CD8^+^ T cells after anti–PD-1 therapy were associated with a poor prognosis. **(A)** Schematic illustration of the schedule for peripheral blood collection of NSCLC patients undergoing anti-PD-1 therapy (*N = 25*). Blood samples were drawn at baseline and following one course of anti-PD-1 mAb (week 3). Fresh PBMCs were isolated directly after collection and stained for flow cytometry analysis. **(B)** Flow cytometry plots showing the gating strategy to identify the CD8^+^CTSL^+^ cell subsets in PBMCs. **(C, D)** Representative flow cytometry plots **(C)** and the frequency **(D)** of peripheral CD8^+^CTSL^+^ cell subsets were compared before and after therapy in responders and non-responders. **p < 0.01, ***p < 0.001. **(E, F)** CTSL Loss in peripheral CD8^+^ T cells predicted clinical response to anti-PD-1 therapy. Receiver operating characteristic (ROC) curves analysis based on the frequency of peripheral CD8^+^CTSL^+^ cell subsets before **(E)** and after therapy **(F)**. The figure shows, for each timepoint, the best values of sensitivity and specificity to calculate the optimal cut-offs (Youden index associated criterion). AUC, Area Under the Curve ROC. **(G)** Kaplan-Meier curves for PFS stratified by the optimal cutoff (<82.25%) of post-treatment CD8^+^CTSL^+^T cells.

To observe the impact of CTSL expression in CD8^+^ T cells on the clinical response to anti-PD-1 immunotherapy, This study enrolled 25 NSCLC patients and systematically examined the expression of CTSL in peripheral CD8^+^ T cells before treatment and after one course (week 3) (**Figure 1A**). The baseline clinical and demographic characteristics of the patient population have been summarized in **Table 1**. We identified the expression changes of CTSL in CD8^+^ T cells by flow cytometry following the isolation of PBMCs (**Figure 1B**). We found that responders maintained stable CD8^+^CTSL^+^ T cell proportions throughout treatment. In contrast, non-responders had a statistically significant decrease of this particular T cell subset after treatment (**Figures 1C, D**; *p=0.0099*). Although there was no difference in CD8^+^CTSL^+^ T cell proportions between responders and non-responders at baseline, the proportion in non-responders decreased significantly at the time point of post-treatment (**Figure 1D**; *p=0.0006*). We also evaluated the potential of CD8^+^CTSL^+^ T cells as a predictive biomarker for treatment response. Cutoff values represent the percentage (%) of CD8^+^CTSL^+^ T cells within the total CD8^+^ T cell population. However, ROC analysis of baseline measurements showed limited predictive ability (AUC = 0.5625, *p = 0.6205*). The optimal cutoff (>77.25%) achieved perfect sensitivity (100%) but low specificity (35.29%; **Figure 1E**). In contrast, post-treatment measurements showed significantly improved predictive ability. At the optimal cutoff (<82.25%), this approach balanced good sensitivity (87.5%) with strong specificity (88.24%; **Figure 1F**), suggesting that monitoring CD8^+^CTSL^+^ T cells during treatment provides valuable predictive information. Furthermore, based on the optimal cutoff (<82.25%) identified in the post-treatment ROC analysis, we evaluated the prognostic value of CD8^+^CTSL^+^ T cells for PFS. As shown in **Figure 1G**, patients with a post-treatment CD8^+^CTSL^+^ T cell proportion ≥82.25% exhibited significantly prolonged PFS compared to those with proportions below the cutoff (log-rank, *p* < 0.001). These results demonstrate that loss of CTSL expression in peripheral CD8^+^ T cells following anti-PD-1 immunotherapy correlates with resistance and may contribute mechanistically to treatment refractoriness.

**Table 1:** Clinicopathologic characteristics of anti-PD-1 immunotherapy.

Patient characteristics	All (n=25)	Responders (n=17)	Non-responders (n=8)	*P Value*
Age: median(range)	63 (46~81)	61 (46~81)	65 (48~68)	*0.3499*
Sex, no. (%)				*>0.9999*
Male	20 (80.0)	13 (76.5)	7 (87.5)	
Female	5 (20.0)	4 (23.5)	1 (12.5)	
Histology, no. (%)				*0.6396*
Squamous cell carcinoma	18 (72.0)	13 (76.5)	5 (62.5)	
Adenocarcinoma	7 (28.0)	4 (23.5)	3 (37.5)	
Disease stage, no. (%)				*>0.9999*
c-stage III	11 (44.0)	8 (47.1)	3 (37.5)	
c-stage IV	14 (56.0)	9 (52.9)	5 (62.5)	
Response, no. (%)				*<0.0001*
CR	3 (12.0)	3 (17.6)	0 (0)	
PR	12 (48.0)	12 (70.6)	0 (0)	
SD	2 (8.0)	2 (11.8)	0 (0)	
PD	8 (32.0)	0 (0)	8 (100.0)	

**Figure 2:**
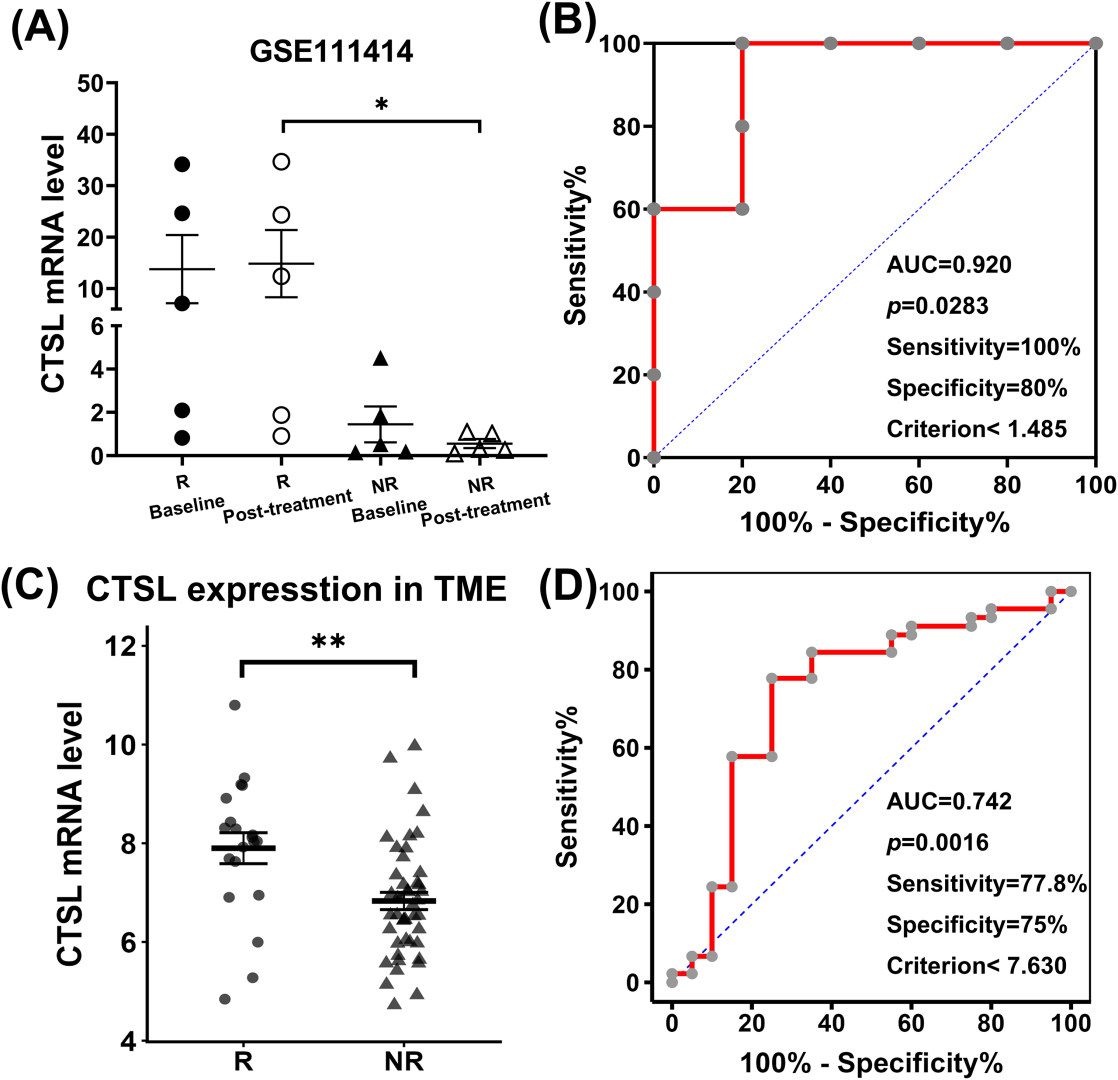
CTSL loss is a biomarker for resistance to anti-PD-1 immunotherapy in peripheral blood and tumor biopsies. **(A)** Comparison of CTSL mRNA expression levels in peripheral CD8^+^ T cells from the GSE111414 dataset. Samples were obtained from responders (n=5) and non-responders (n=5) at baseline and post-treatment (before and after anti-PD-1 therapy, respectively). *p < 0.05. **(B)** ROC curve analysis based on CTSL mRNA expression levels in peripheral CD8^+^ T cells from responders versus non-responders after therapy. **(C)** Comparison of CTSL mRNA expression levels in NSCLC tumor biopsies from the merged cohort (GSE126044, GSE135222, GSE166449). **P < 0.01. TME, tumor microenvironment. **(D)** The ROC curve of CTSL in predicting the response status of anti-PD-1 therapy based on the merged cohort.

To further validate the association between CTSL loss and resistance to anti-PD-1 therapy, this study conducted bioinformatics validation analyses using sequencing data from both peripheral CD8^+^ T cells and tumor biopsy samples. Examination of the public dataset GSE111414 revealed that CTSL mRNA expression in peripheral CD8^+^ T cells remained stable in responders after anti-PD-1 treatment, whereas non-responders showed a significant reduction in this measure (**Figure 2A**; *p < 0.05*). ROC curve analysis based on post-treatment CTSL mRNA levels demonstrated its potential to discriminate response status (AUC = 0.920, *p = 0.0283*), with an optimal cutoff (<1.485) yielding perfect sensitivity (100%) and high specificity (80%; **Figure 2B**). Furthermore, integration of multiple NSCLC biopsy sequencing datasets (GSE126044, GSE135222, GSE166449) indicated that CTSL expression was similarly down-regulated in the TME of non-responders (**Figure 2C**; *p < 0.01*), and this metric exhibited considerable discriminative power in predicting therapeutic outcome (AUC = 0.742, *p = 0.0016*), achieving high sensitivity (77.8%) and specificity (75%) at the optimal cutoff (<7.630; **Figure 2D**). Collectively, these findings support that loss of CTSL expression in both peripheral CD8^+^ T cells and tumor tissues serves as a cross-dimensional biomarker for resistance to anti-PD-1 immunotherapy.

### The changes of distribution and CTSL expression in different T-cell subsets from responders or non-responders

**Figure 3:**
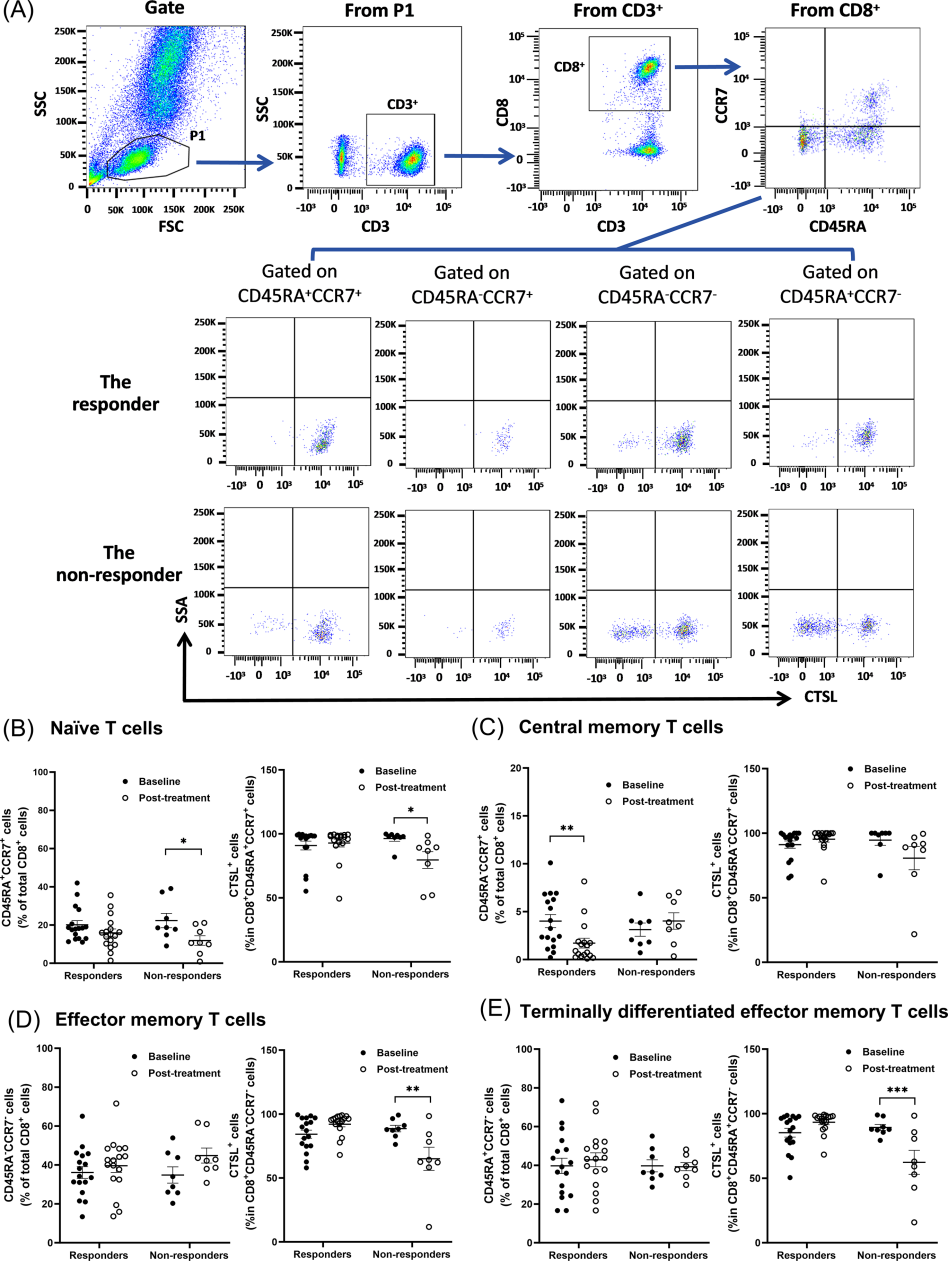
The distribution and CTSL expression of different T-cell subsets from responders or nonresponders. **(A)** Representative flow cytometry plots are shown illustrating the gating strategy for CTSL expression in T-cell subsets. Representative dot plots of CCR7/CD45RA subsets are shown for one responder and one non-responder. **(B–E)** Peripheral blood was prospectively collected from patients with NSCLC before (baseline) and after anti-PD-1 mAb (week 3). Responders and nonresponders were compared for alterations in the frequencies of naïve T cells **(B)**, central memory T cells **(C)**, effector memory T cells **(D)**, and terminally differentiated effector memory T cells **(E)**. The dynamic changes in the CTSL^+^ cell subsets within these four T-cell subsets were further analyzed. *p < 0.05, **p < 0.01, ***p < 0.001.

To further validate our findings, we characterized the phenotypic differences in CTSL-expressing CD8^+^ T cells between responders and non-responders by conducting immunophenotypic profiling of T-cell subsets using CCR7 and CD45RA surface markers (**Figure 3A**). The immunophenotypic stratification of CD8^+^ T cell subsets was as follows: naïve (NT: CD45RA^+^CCR7^+^), central memory (TCM: CD45RA^-^CCR7^+^), effector memory (TEM: CD45RA^-^CCR7^-^), and terminally differentiated effector memory (TEMRA: CD45RA^+^CCR7^-^) subsets. We first assessed the longitudinal changes in the proportion of these subsets within peripheral CD8^+^ T cells from responders and non-responders, at baseline and post-treatment (**Figures 3B–E**). Following the first course of anti-PD-1 immunotherapy, non-responders exhibited a decreased proportion of NT subsets and responders exhibited an decreased proportion of TCM subsets within peripheral CD8^+^ T cells (**Figures 3B, C**). Meanwhile, no significant changes were observed in TEM or TEMRA subset proportions relative to baseline (**Figures 3D, E**). Further research has found that a subset-specific pattern of CTSL regulation in non-responders. When we examined different CD8^+^ T cell subsets, the NT subset showed a slight but statistically significant decrease in CTSL after treatment (*p=0.0169*), while TCM cells maintained relatively stable expression levels (**Figures 3B, C**, *p>0.05*). More notably, both TEM and TEMRA subsets demonstrated substantial reductions in CTSL after therapy (**Figures 3D, E**; *p=0.0014* and *p=0.0004*, respectively). Considering the crucial contributions of TEM and TEMRA cells to anti-tumor immunity, their selective loss of CTSL strongly implies that this protease participates in the molecular pathways that determine treatment resistance.

### CTSL enhances cytotoxic function and proliferation of peripheral CD8^+^ T cells

**Figure 4:**
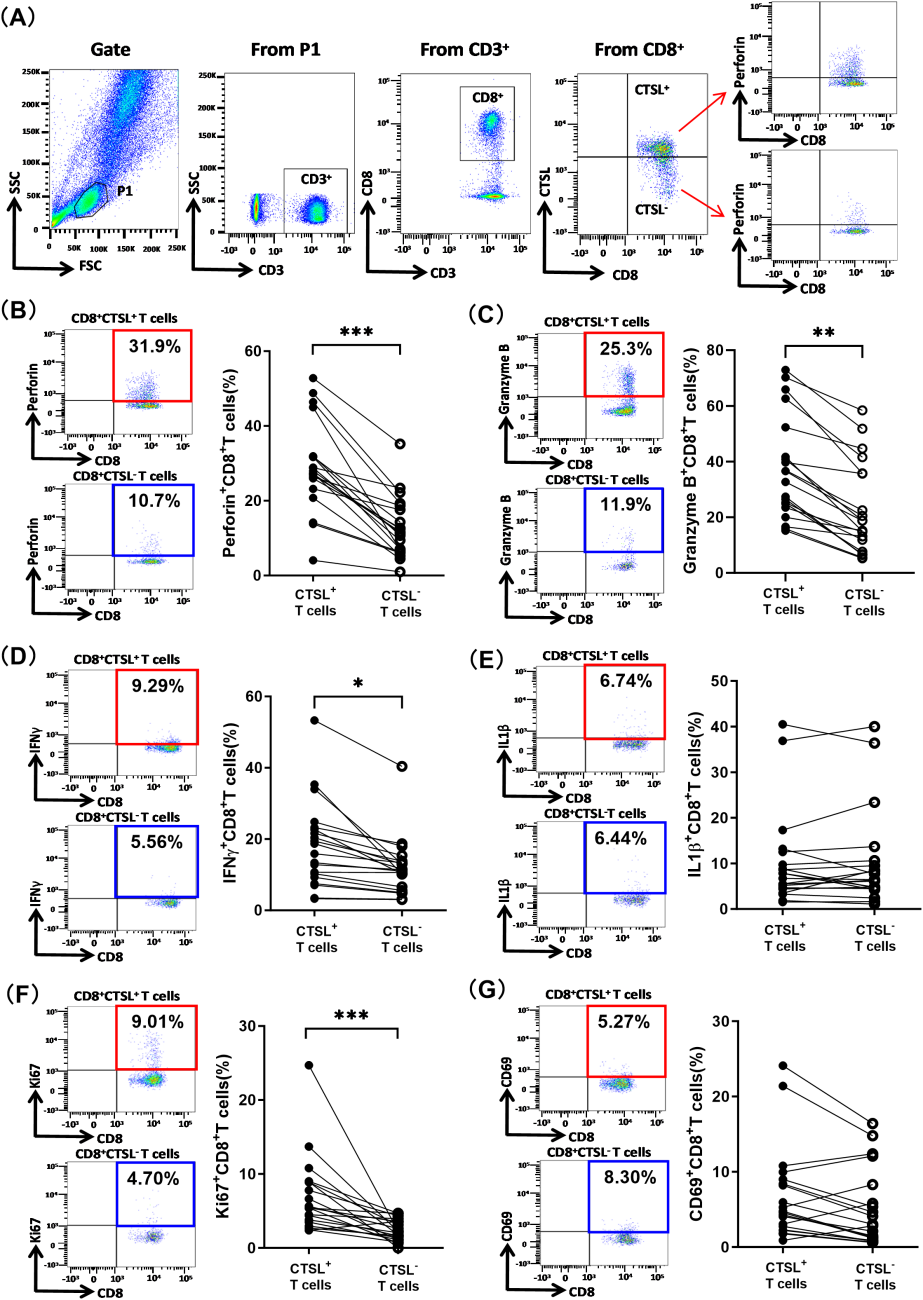
CTSL enhances the cytotoxic function and proliferative capacity of CD8^+^ T cell. **(A)** Representative flow cytometry plots illustrating the gating strategy employed to evaluate functional molecule expression in peripheral CD8^+^ T cells stratified by CTSL expression levels. **(B–G)** Representative flow cytometry plots and quantitative data depicting expression levels of perforin **(B)**, granzyme B **(C)**, IFN-γ **(D)**, IL-1β **(E)**, Ki67 **(F)**, and CD69 **(G)** in CD8^+^ T cells stratified by CTSL expression levels. Samples were derived from 20 NSCLC patients after completing the first course (week 3) of anti-PD-1 therapy. Bars indicate mean with standard error of mean (± SEM). *p < 0.05, **p < 0.01, ***p < 0.001.

To assess how CTSL influences the anti-tumor response of peripheral CD8^+^ T cells, we analyzed the expression of key functional molecules (perforin, granzyme B, IFN-γ, IL-1β, Ki67, and CD69), grouping the cells by CTSL expression levels. After the first course of anti-PD-1 immunotherapy, PBMCs from 20 NSCLC patients were collected and analyzed by flow cytometry. The gating strategy was presented in **Figure 4A**. We found that peripheral CD8^+^CTSL^+^ T cells contained higher expression of perforin (*p < 0.001*) and granzyme B (*p < 0.01*) than CD8^+^CTSL^-^ T cells (**Figures 4B, C**). Cytokine analysis further found that IFN-γ expression was significantly increased in CD8^+^CTSL^+^ T cells (**Figure 4D**, *p < 0.05*) but IL-1β levels remained unchanged (**Figure 4E**, *p > 0.05*). Moreover, CD8^+^CTSL^+^ T cells showed heightened expression of the proliferation marker Ki67 (**Figure 4F**, *p < 0.001*), whereas no significant alteration was observed in the tissue-residency marker CD69 (**Figure 4G**, *p = 0.12*). These results confirm that CTSL promotes both the cytotoxic activity and proliferation of peripheral CD8^+^ T cells.

### Expression correlation and functional validation of CTSL and NLRP3 in peripheral CD8^+^ T cells

**Figure 5:**
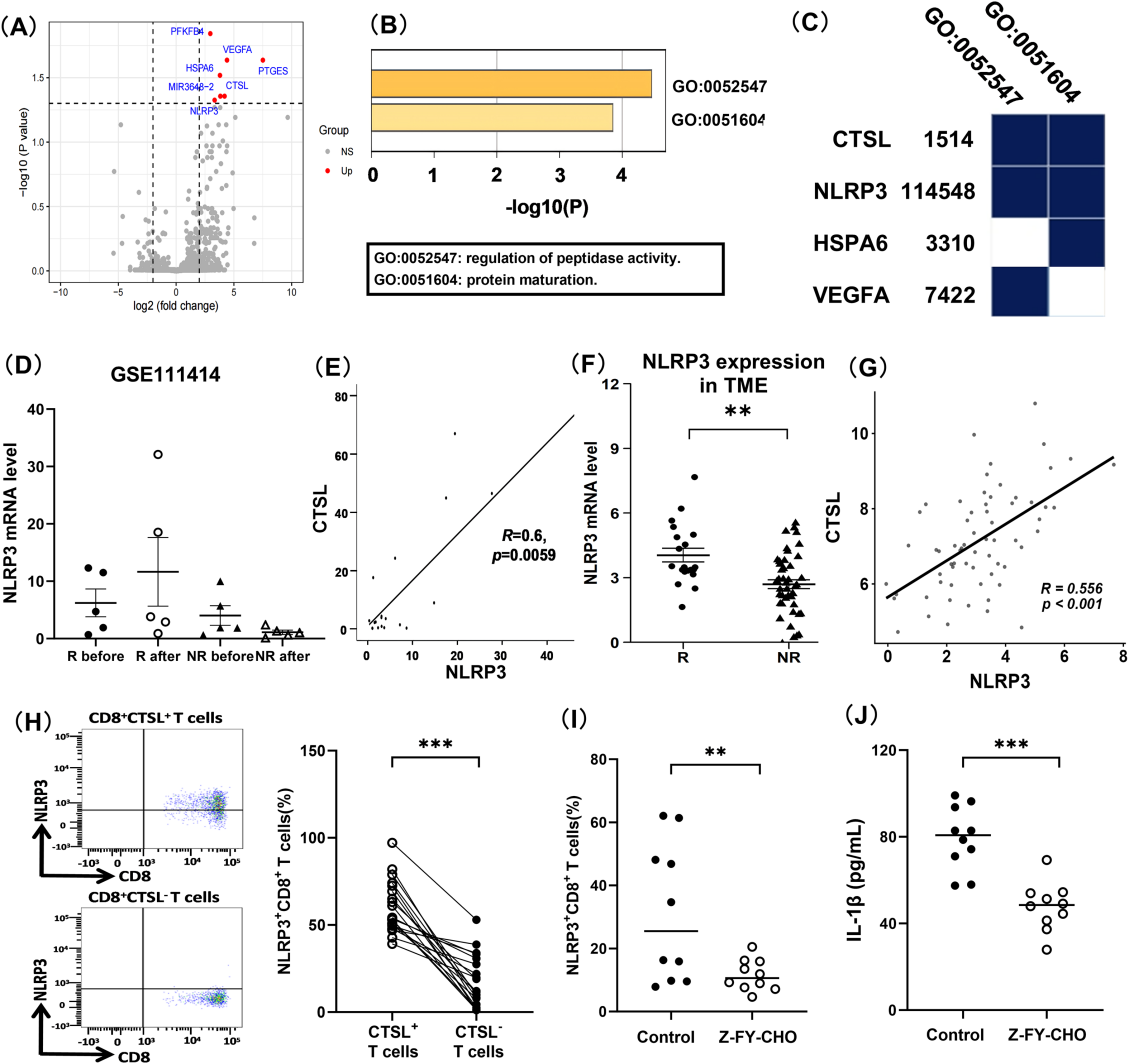
Expression correlation and functional validation of CTSL and NLRP3 in peripheral CD8^+^ T cells. **(A)** Volcano plot of differentially expressed genes in peripheral CD8^+^ T cells between responders and non-responders following anti-PD-1 therapy from the GSE111414 dataset. Red dots represent differentially expressed genes with log2 fold change >1, and grey spots represent genes with no significant difference in expression, adjusted p-value <0.05. **(B)** GO enrichment analysis of differentially expressed genes in R group and NR group by Metascape. **(C)** Heatmaps of these differentially expressed genes associated with the two best-scoring GO groups. **(D)** The comparison of NLRP3 mRNA expression levels in peripheral CD8^+^ T cells from responders (n=5) and non-responders (n=5), collected before and after anti-PD-1 therapy. R before: responders before treatment; R after: responders after treatment; NR before: non-responders before treatment; NR after: non-responders after treatment. **(E)** Correlation between CTSL mRNA expression and NLRP3 mRNA expression in peripheral CD8^+^ T cells from the GSE111414; the X-axis represents the NLRP3 mRNA expression and the Y-axis shows the CTSL mRNA expression. **(F)** The comparison of NLRP3 mRNA expression levels in NSCLC tumor biopsies from the merged cohort (GSE126044, GSE135222, GSE166449). TME: tumor microenvironment. **(G)** Correlation between CTSL mRNA expression and NLRP3 mRNA expression in NSCLC biopsies from the merged cohort. the X-axis represents the NLRP3 mRNA expression and the Y-axis shows the CTSL mRNA expression. **(H)** Representative flow cytometry plots and quantitative data depicting expression levels of NLRP3 in CD8^+^ T cells stratified by CTSL expression levels. Samples were derived from 20 NSCLC patients after completing the first course (week 3) of anti-PD-1 therapy. Bars indicate mean with standard error of mean (± SEM). *p < 0.05, **p < 0.01, ***p < 0.001. **(I, J)** PBMCs from NSCLC patients (n=10) were cultured for 24 h with or without the CTSL inhibitor Z-FH-CHO. Subsequent analyses included: **(I)** flow cytometry to quantify the frequency of NLRP3^+^CD8^+^ T cell subsets, and **(J)** ELISA to measure IL-1β levels in culture supernatants. **p < 0.01, ***p < 0.001.

To further investigate the mechanism of immunotherapy resistance induced by CTSL loss in peripheral CD8^+^ T cells, we examined the GSE111414 dataset from the GEO database. In that study, PBMCs were prospectively collected from NSCLC patients before and during anti-PD-1 therapy, and mRNA-sequencing analysis was performed on peripheral CD8^+^ T cells isolated from 5 responders and 5 non-responders. The package “DESeq2” was used for differential analysis to obtain the resistance-associated differential genes in the transcriptomic data. Volcano-plot analysis revealed just seven significantly altered genes (including CTSL) in peripheral CD8^+^ T cells between responders and non-responders following anti-PD-1 immunotherapy (**Figure 5A**). The GO enrichment analysis results demonstrated that the differential genes mainly got involved in regulation of peptidase activity and protein maturation (**Figure 5B**). Heatmaps of differentially expressed genes revealed that only CTSL and NLRP3 were simultaneously assigned to both the “regulation of peptidase activity” and “protein maturation” functional categories (**Figure 5C**). Further analysis revealed that in peripheral CD8^+^ T cells, NLRP3 transcript levels showed an upward trend in responders to anti-PD-1 therapy, but a downward trend in non−responders (**Figure 5D**). Consistent with this, a significant positive correlation was observed between CTSL and NLRP3 expression in these cells (**Figure 5E**, R = 0.60, *p=0.0059*). We also examined tumor tissue mRNA expression from NSCLC patients treated with anti-PD-1 therapy using the GEO datasets GSE135222, GSE126044, and GSE166449, along with corresponding clinical information. NLRP3 was significantly upregulated in tumor tissues from responders compared to non−responders (**Figure 5F**, *p<0.01*). Moreover, expression of CTSL and NLRP3 remained positively correlated in tumor tissues (**Figure 5G**, R = 0.556, *p<0.001*). These observations prompted us to further investigate the functional relationship between CTSL and NLRP3.

We validated the functional link between CTSL and NLRP3 through a series of targeted experiments. First, based on CTSL expression levels in CD8^+^ T cells, these cells were divided into CTSL^+^ and CTSL^-^ groups. Flow cytometry analysis revealed that NLRP3 expression levels were significantly higher in CD8^+^CTSL^+^T cells than in CD8^+^CTSL^-^ T cells (**Figure 5H**, *p < 0.001*). Then, using the specific CTSL inhibitor Z-FY-CHO on patient-derived PBMCs, we found that blocking CTSL activity for 24 hours led to a marked decrease in NLRP3 within CD8^+^ T cells (**Figure 5I**, *p < 0.01*). Finally, we extended this observation to pathway function, demonstrating that CTSL inhibition also reduced the secretion of IL-1β, a key NLRP3-dependent cytokine (**Figure 5J**, *p < 0.001*). Therefore, our data suggest a functional association between CTSL and NLRP3 inflammasome activation, suggesting that CTSL may affect the anti-tumor function of CD8^+^ T cells by regulating NLRP3.

### Activation of NLRP3 reverses anti-tumor dysfunction of CD8^+^ T cells induced by CTSL inhibition

**Figure 6:**
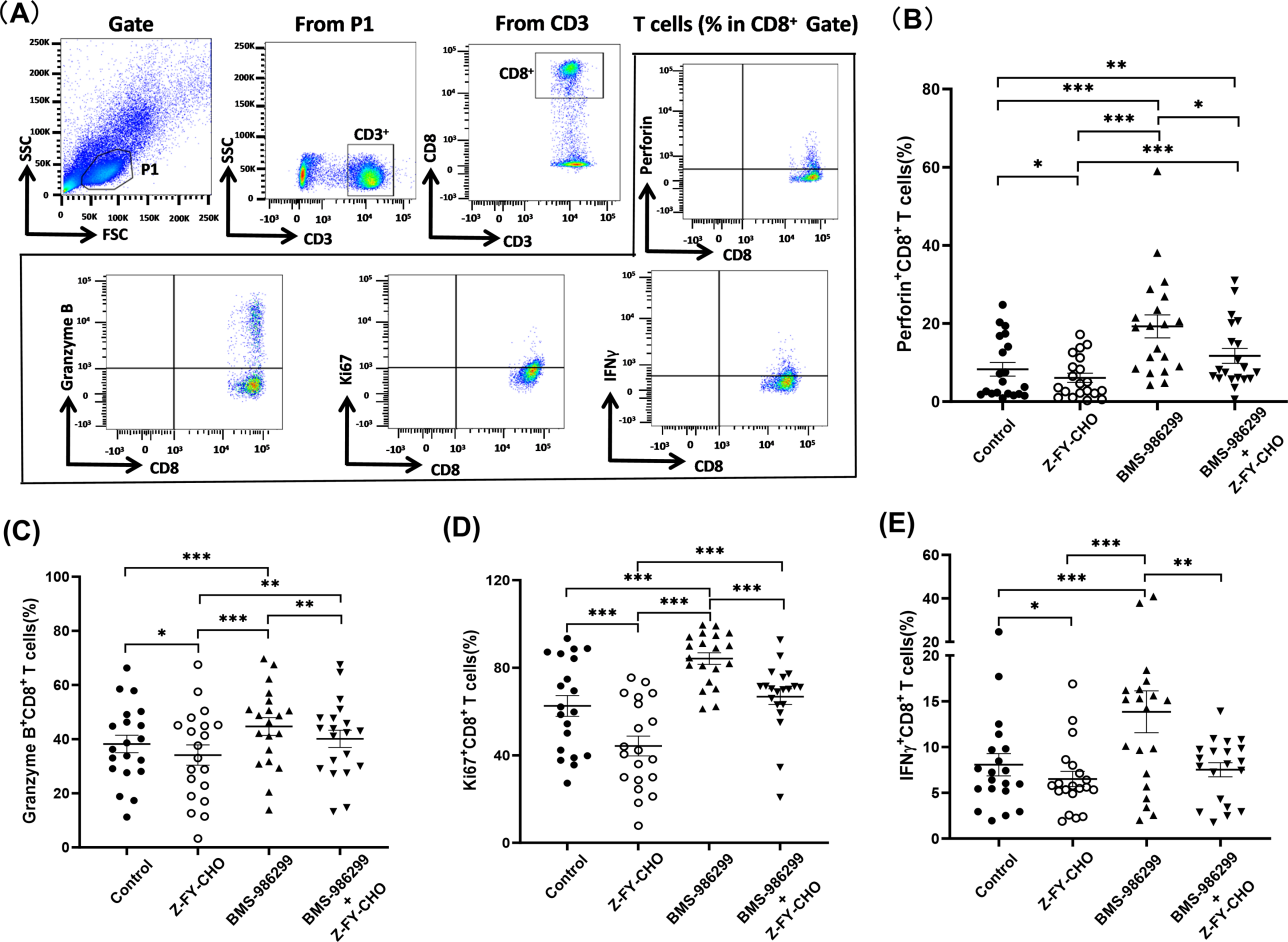
Activation of NLRP3 reverses anti-tumor dysfunction of CD8^+^ T cells induced by CTSL inhibition. **(A)** Representative flow cytometry plots illustrating the gating strategy employed to evaluate functional molecule expression in CD8^+^ T cells. **(B–H)** The expression levels of perforin **(B)**, granzyme B **(C)**, Ki67 **(D)**, and IFN-γ **(E)** in CD8^+^ T cells were compared under four conditions: 1) Control, 2) the CTSL inhibitor Z-FY-CHO (40uM) 3) the NLRP3 agonist BMS-986299(20uM), 4) Z-FY-CHO(40uM) + BMS-986299(20uM). Samples were derived from 20 NSCLC patients. Bars indicate mean with standard error of mean (± SEM). *p < 0.05, **p < 0.01, ***p < 0.001.

To investigate whether CTSL enhances the anti-tumor function of CD8^+^ T cells by activating the NLRP3 inflammasome, we conducted pharmacological intervention experiments using PBMCs from NSCLC patients. The experimental groups included vehicle control (DMSO), the CTSL inhibitor Z-FY-CHO (mimicking CTSL loss in PBMCs from anti-PD-1-resistant patients), the NLRP3 agonist BMS-986299, and the combination of Z-FY-CHO and BMS-986299 (to assess whether NLRP3 activation could reverse the effects of CTSL loss, thereby demonstrating that CTSL functions through the NLRP3 inflammasome). After 24 hours of treatment, the expression levels of effector molecules (perforin, granzyme B, Ki67, IFN-γ) in CD8^+^ T cells were measured by multicolor flow cytometry (**Figure 6A**).

The experimental results showed that treatment with the CTSL inhibitor Z-FY-CHO alone significantly reduced the expression levels of perforin (*p < 0.05*), granzyme B (*p < 0.05*), the proliferation marker Ki67 (*p < 0.001*), and IFN-γ (*p < 0.05*) in CD8^+^ T cells (**Figures 6B–E**). In contrast, treatment with the NLRP3 agonist BMS-986299 alone significantly upregulated the expression of these effector molecules. In the combination treatment group, BMS-986299 significantly reversed the inhibitory effects of Z-FY-CHO on perforin (*p < 0.001*), granzyme B (*p < 0.01*), and Ki67 (*p < 0.001*). The suppression of IFN-γ also showed a trend toward reversal, although it did not reach statistical significance. These findings demonstrate that activating NLRP3 can reverse the anti-tumor dysfunction of CD8^+^ T cells induced by CTSL inhibition, suggesting that CTSL may enhance the anti-tumor function of CD8^+^ T cells through activation of the NLRP3 inflammasome.

**Figure 7:**
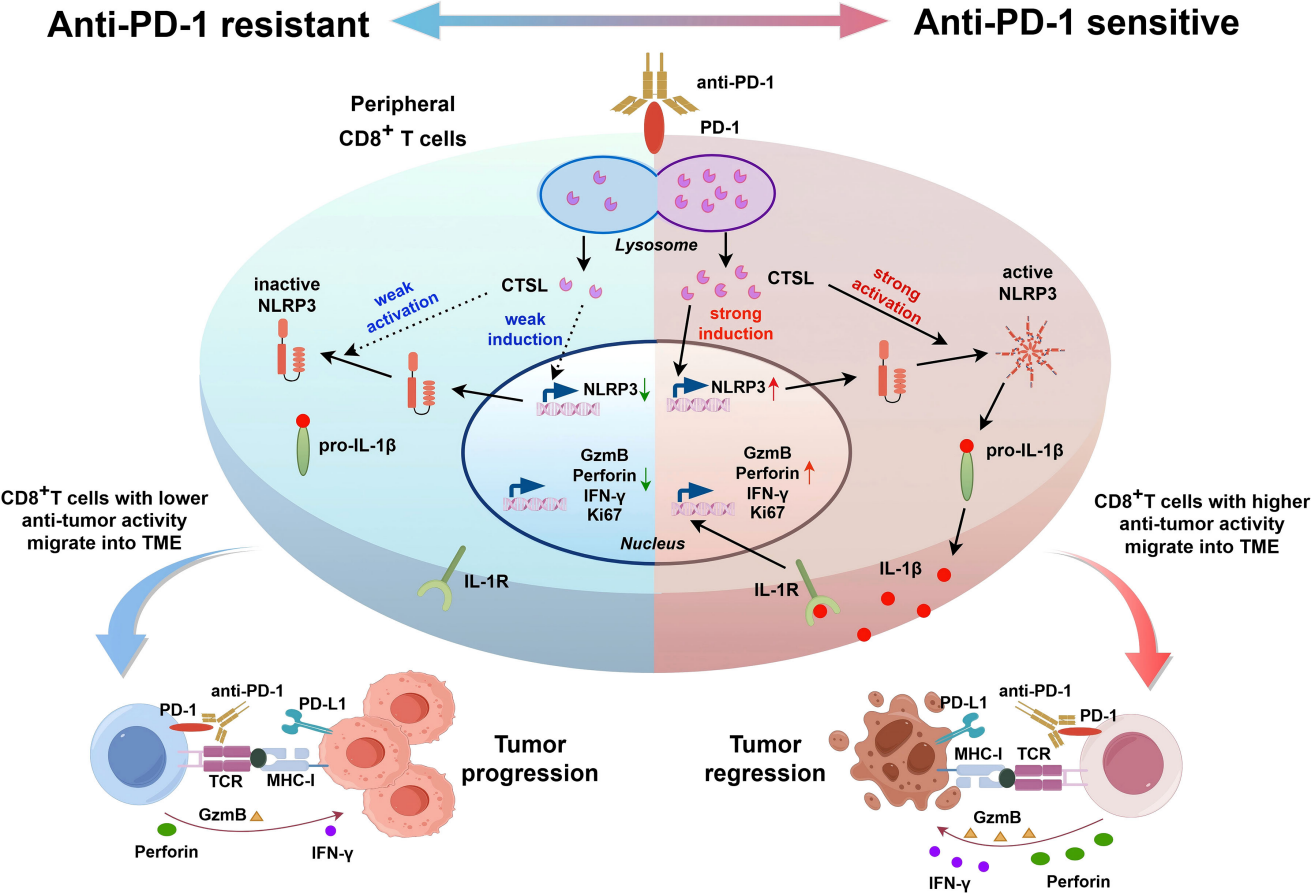
Schematic model showing the mechanism of CTSL-NLRP3 axis for enhancing anti‐PD‐1 therapeutic efficacy in NSCLC. CTSL expressed in peripheral CD8^+^ T cells promotes the expression of NLRP3 and activates the NLRP3 inflammasome, thereby inducing the autocrine secretion of the effector molecule IL-1β. Subsequently, the autocrine IL-1β activates the IL-1R signaling pathway, stimulating the expression of effector molecules (such as perforin, granzyme, and IFN-γ) and promoting cell proliferation (as indicated by Ki67). These highly active peripheral CD8^+^ T cells migrate into the TME, enhancing the efficacy of anti-PD-1 therapy in NSCLC. Conversely, the absence of CTSL expression in peripheral CD8^+^ T cells leads to resistance to anti-PD-1 treatment in NSCLC. Schematic model is created using Figdraw (www.figdraw.com).

Based on our findings, we propose a mechanistic model in which CTSL enhances the anti-tumor function of CD8^+^ T cells by activating the NLRP3 inflammasome. In peripheral CD8^+^ T cells, CTSL not only upregulates the transcriptional level of NLRP3 but also promotes the activation of the NLRP3 inflammasome, thereby forming a synergistic activation loop that enhances the anti-tumor function of CD8^+^ T cells. These highly active peripheral CD8^+^ T cells, after migrating into the TME, improve the efficacy of anti-PD-1 therapy in NSCLC. Conversely, loss of CTSL expression in peripheral CD8^+^ T cells leads to resistance to anti-PD-1 therapy in NSCLC (**Figure 7**).

## Discussion

Based on our observations, patients who responded to treatment maintained a stable proportion of CD8^+^CTSL^+^ T cells throughout the therapy (**Figure 1C**), whereas resistant patients exhibited a marked decline in this subset following treatment (**Figure 1D**). We propose that this phenomenon may be attributed to the following mechanisms. First, anti-PD-1 antibodies that recognize the membrane-proximal region of the PD-1 molecule may cross-link PD-1 and thereby trigger inhibitory signals in T cells (29). Given that the binding epitopes of such antibodies on PD-1 vary among patients, this may impede effective activation of CD8^+^ T cells in some individuals, leading to downregulation of CTSL expression. Our results show that CTSL expression levels are significantly lower in CD8^+^ TEM and TEMRA cells than in other subsets (**Figure 3**). Previous studies have reported that PD-1 expression is markedly higher in CD8^+^ TEM and TEMRA cells compared to CD8^+^ NT and TCM cells (30). When anti-PD-1 antibodies bind to PD-1 on these two subsets, the greater spatial hindrance may increase the likelihood of epitope binding variability. Second, the IgG4_S228P_ heavy chain of clinically used anti-PD-1 antibodies (e.g., nivolumab and pembrolizumab) retains Fc gamma receptor (FcγR) binding capacity (31). PD-1 antibodies inhibit T cell activation in an FcγR-dependent manner (32). Abnormal interactions between PD-1 antibodies and FcγR in patients may suppress T cell activation signaling, thereby reducing CTSL levels. Finally, lysosomal dysfunction caused by various factors (e.g., pharmacological agents, physiological conditions, or pathological states) may contribute to CTSL down-regulation (33–35).

Our findings demonstrate that CTSL expression in peripheral CD8^+^ T cells is positively correlated with both the expression level and activation status of NLRP3 (**Figures 5E, H–J**). Current evidence indicates that NLRP3 inflammasome activation typically requires two key biological processes: transcriptional induction of NLRP3 expression and post-translational inflammasome assembly (36). Yang et al. reported that in human glioma cells, CTSL acts as an upstream activator of NF-κB, a signaling pathway well established as a critical regulator of NLRP3 transcription (36–39). Therefore, CTSL may promote NLRP3 transcription via the NF-κB signaling pathway. Supporting this notion, previous studies have shown that CTSL can activate TLR9 through proteolytic cleavage, thereby initiating NF-κB signaling and upregulating NLRP3 expression (40, 41). In addition to enhancing transcriptional regulation of NLRP3, CTSL can also directly promote NLRP3 inflammasome assembly (42, 43). Activated NLRP3 inflammasome further promotes IL-1β secretion (44). To investigate whether CTSL loss restricts NLRP3 inflammasome assembly, we performed ELISA detection of IL-1β in culture supernatants from each group in the pharmacological intervention experiment. The results showed that treatment with the CTSL inhibitor Z-FY-CHO significantly suppressed the secretion of IL-1β in PBMCs of NSCLC patients, whereas treatment with the NLRP3 agonist BMS-986299 significantly enhanced IL-1β secretion (**Supplementary Figure 1**). Furthermore, we found that CTSL expression levels in peripheral CD8^+^ T cells had no significant impact on IL-1β expression itself (**Figure 4E**). Collectively, these findings suggest that CTSL loss primarily impairs NLRP3 inflammasome assembly, leading to defective IL-1β secretion rather than affecting IL-1β expression. This result further supports the notion that CTSL regulates the anti-tumor function of CD8^+^ T cells by modulating NLRP3 inflammasome assembly. Based on the literature and our findings, we propose that CTSL not only increases the transcriptional expression level of NLRP3 but also activates the physical assembly of the NLRP3 inflammasome, thereby forming a synergistic activation loop that enhances the anti-tumor function of CD8^+^ T cells (**Figure 7**). This model provides novel molecular insights into how CTSL shapes anti-tumor immunity.

Functional experiments revealed that the NLRP3 agonist enhanced the anti-tumor function of peripheral CD8^+^ T cells and reversed the anti-tumor dysfunction induced by CTSL inhibition (**Figure 6**). This is consistent with previous reports demonstrating that the NLRP3-IL-1β axis enhances T cell cytotoxicity, highlighting the important role of the NLRP3 inflammasome in regulating CD8^+^ T cell activity (45–47). Several studies have also confirmed that pharmacological inhibition of NLRP3 inflammasome activity in CD8^+^ T cells reduces CD8^+^ T cell activity in autoimmune or infectious diseases (48, 49). Furthermore, it has been reported that inactivation of the NLRP3 inflammasome in CD8^+^ T cells promotes tumor progression (50). Nelson BE et al. reported that the NLRP3 agonist BMS-986299, when combined with anti-PD-1 monotherapy, significantly promoted T cell infiltration into the TME and enhanced the efficacy of anti-PD-1 immunotherapy (51). Kim DK et al. also found that Denfivontinib activated effector CD8^+^ T cells via the NLRP3 inflammasome and, in combination with anti-PD-1 monotherapy, produced potent anti-cancer effects (52). These studies further suggest that activating the NLRP3 inflammasome enhances anti-PD-1 immunotherapy by promoting T cell function. However, some investigators have found that CD8^+^ T cell function remains normal in patients with hyperactive NLRP3, suggesting that although CD8^+^ T cells indeed express NLRP3, normal human CD8^+^ T cell activity does not require the canonical complement signaling-activated NLRP3 inflammasome (53). These findings suggest that the activation and function of CD8^+^ T cells by the NLRP3 inflammasome are highly dependent on the cellular context and pathological state, and that its promoting effects may occur through non-canonical pathways or become more prominent only under specific pathological conditions (e.g., tumors, autoimmune diseases). Future studies are needed to elucidate this complex regulatory mechanism.

In conclusion, loss of CTSL expression in peripheral CD8^+^ T cells following anti-PD-1 immunotherapy predicts resistance to anti-PD-1 treatment in patients with NSCLC. CTSL loss may suppress the anti-tumor function of CD8^+^ T cells by inhibiting NLRP3 inflammasome activation. This study not only provides a potential circulating biomarker for predicting response to anti-PD-1 therapy but also offers new insights into the mechanisms underlying immunotherapy resistance.

## Data Availability

The original contributions presented in the study are included in the article/**Supplementary Material**. Further inquiries can be directed to the corresponding author.

## Supplementary material

Supplementary Figure 1 IL1β levels in PBMC culture supernatant of NSCLC patients. PBMCs from NSCLC patients (n=6) were cultured for 24 h under four conditions: 1) Control, 2) the CTSL inhibitor Z-FY-CHO 3) the NLRP3 agonist BMS-986299, 4) Z-FY-CHO + BMS-986299. ELISA to measure IL-1β levels in culture supernatants. Student’;s paired t-test, *P < 0.05, **P < 0.01, ***P < 0.001.
