# Supplementary figures and images for: CTSL loss leads to anti-PD-1 immunotherapy resistance in lung cancer by suppressing the anti-tumor function of peripheral CD8+ T cells
Source: Front Immunol. 2026 Jun 10;17:1863563. doi: 10.3389/fimmu.2026.1863563 (PMC13290973; doi:10.3389/fimmu.2026.1863563)

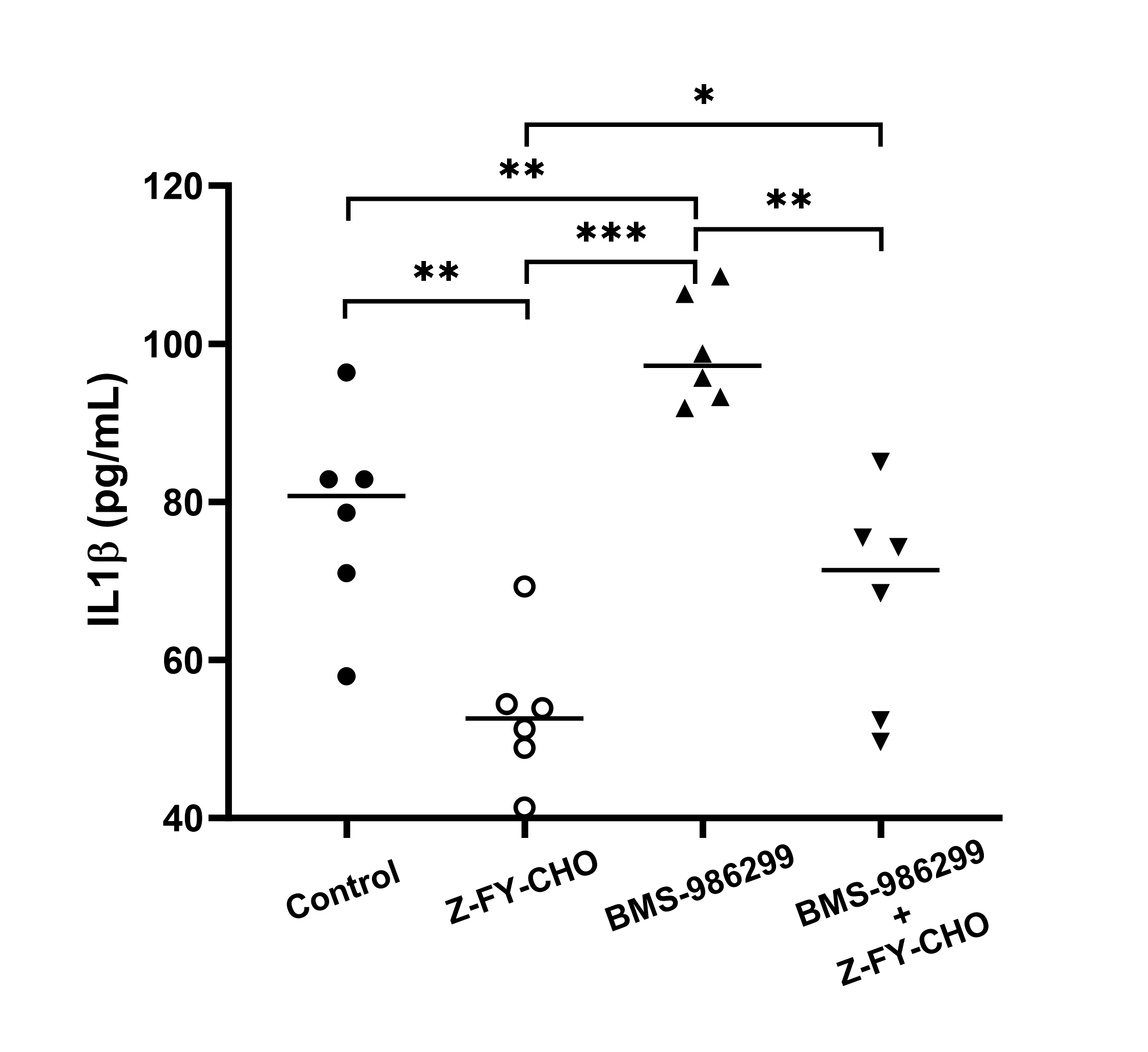

Supplement: Supplementary Figure 1 — IL1β levels in PBMC culture supernatant of NSCLC patients. PBMCs from NSCLC patients (n=6) were cultured for 24 h under four conditions: 1) Control, 2) the CTSL inhibitor Z-FY-CHO 3) the NLRP3 agonist BMS-986299, 4) Z-FY-CHO + BMS-986299. ELISA to measure IL-1β levels in culture supernatants. Student’;s paired t-test, *P < 0.05, **P < 0.01, ***P < 0.001. [file Image1.tif]
