# Supplementary material for: CTSL loss leads to anti-PD-1 immunotherapy resistance in lung cancer by suppressing the anti-tumor function of peripheral CD8+ T cells
Source: Front Immunol. 2026 Jun 10;17:1863563. doi: 10.3389/fimmu.2026.1863563 (PMC13290973; doi:10.3389/fimmu.2026.1863563)
Supplement: Supplementary file 2 [file Table1.docx]

**Table S1 - Overview of all antibodies used for the actice.**

| **ANTIBODIES** | **COMPANY** | **IDENTIFIER** |
| --- | --- | --- |
| Anti-human CD3 (mouse, clone UCHT1 ),  FITC conjugated | BD Biosciences | Cat# 555916, RRID:AB_396217 |
| Anti-human CD3 (mouse, clone OKT3),  BV510 conjugated | BD Biosciences | Cat# 566780, RRID:AB_2869862 |
| Anti-human CD8 (mouse, clone RPA-T8), PE-Cy7 conjugated | BD Biosciences | Cat# 557746,  RRID:AB_396852 |
| Anti-human CD45RA (mouse, clone HI10), BV605conjugated | BD Biosciences | Cat# 562886, RRID:AB_2737865 |
| Anti-human CD197 (mouse, clone 150503), PerCP-Cy5.5conjugated | BD Biosciences | Cat# 561144, RRID:  AB_10562553 |
| Anti-human CD69 (mouse, clone FN50 ), Alexa Fluor 700 conjugated | BD Biosciences | Cat# 560739, RRID:AB_1727505 |
| Anti-human IFN-γ (mouse, clone 4S.B3), PerCP-Cy5.5 conjugated | BD Biosciences | Cat# 560742,  RRID:AB_1727531 |
| Anti-human Perforin (mouse, clone δG9),  FITC conjugated | BD Biosciences | Cat# 567722, RRID:AB_2916709 |
| Anti-human Ki-67 (mouse, clone Ki-67),  PE conjugated | BD Biosciences | Cat# 570923, RRID:AB_3686113 |
| Anti-human Granzyme B (mouse, clone GB11), Alexa Fluor 647 conjugated | BD Biosciences | Cat# 561999, RRID:AB_10897997 |
| Anti-human Granzyme B (mouse, clone GB11), PE conjugated | BD Biosciences | Cat# 561142, RRID:AB_10561690 |
| Anti-human IL-1β (mouse, clone 8516),  PE conjugated | R&D Systems | Cat# IC201P,  RRID:AB_357275 |
| Anti-human NLRP3 (mouse, clone 652519), Alexa Fluor 700 conjugated | R&D Systems | Cat# IC67892N,  RRID:AB_3656419 |
| Anti-human CTSL (mouse, clone 33/2),  Alexa Fluor 647 conjugated | Santa Cruz Biotechnology | Cat# sc-32320,  RRID:AB_626811 |
